# The Effects of Cold Exposure on Leukocytes, Hormones and Cytokines during Acute Exercise in Humans

**DOI:** 10.1371/journal.pone.0110774

**Published:** 2014-10-22

**Authors:** Dominique D. Gagnon, Sheila S. Gagnon, Hannu Rintamäki, Timo Törmäkangas, Katri Puukka, Karl-Heinz Herzig, Heikki Kyröläinen

**Affiliations:** 1 Institute of Biomedicine, Department of Physiology and Biocenter of Oulu, University of Oulu, Oulu, Finland; 2 Department of Biology of Physical Activity, University of Jyväskylä, Jyväskylä, Finland; 3 Department of Health and Rehabilitation Sciences, University of Western Ontario, London, Canada; 4 Finnish Institute of Occupational Health, Oulu, Finland; 5 Department of Health Sciences, University of Jyväskylä, Jyväskylä, Finland; 6 NordLab Oulu, Oulu University Hospital and Department of Clinical Chemistry, University of Oulu, Oulu, Finland; 7 Medical Research Center Oulu and Oulu University Hospital, Oulu, Finland; University of Bern, Switzerland

## Abstract

The purpose of the study was to examine the effects of exercise on total leukocyte count and subsets, as well as hormone and cytokine responses in a thermoneutral and cold environment, with and without an individualized pre-cooling protocol inducing low-intensity shivering. Nine healthy young men participated in six experimental trials wearing shorts and t-shirts. Participants exercised for 60 min on a treadmill at low (LOW: 50% of peak VO_2_) and moderate (MOD: 70% VO_2peak_) exercise intensities in a climatic chamber set at 22°C (NT), and in 0°C (COLD) with and without a pre-exercise low-intensity shivering protocol (SHIV). Core and skin temperature, heart rate and oxygen consumption were collected continuously. Blood samples were collected before and at the end of exercise to assess endocrine and immunological changes. Core temperature in NT was greater than COLD and SHIV by 0.4±0.2°C whereas skin temperature in NT was also greater than COLD and SHIV by 8.5±1.4°C and 9.3±2.5°C respectively in MOD. Total testosterone, adenocorticotropin and cortisol were greater in NT *vs.* COLD and SHIV in MOD. Norepinephrine was greater in NT *vs.* other conditions across intensities. Interleukin-2, IL-5, IL-7, IL-10, IL-17, IFN-γ, Rantes, Eotaxin, IP-10, MIP-1β, MCP-1, VEGF, PDGF, and G-CSF were elevated in NT *vs.* COLD and/or SHIV. Furthermore, IFN-γ, MIP-1β, MCP-1, IL-10, VEGF, and PDGF demonstrate greater concentrations in SHIV *vs.* COLD, mainly in the MOD condition. This study demonstrated that exercising in the cold can diminish the exercise-induced systemic inflammatory response seen in a thermoneutral environment. Nonetheless, prolonged cooling inducing shivering thermogenesis prior to exercise, may induce an immuno-stimulatory response following moderate intensity exercise. Performing exercise in cold environments can be a useful strategy in partially inhibiting the acute systemic inflammatory response from exercise but oppositely, additional body cooling may reverse this benefit.

## Introduction

The immunological and endocrine systems, now described as one bi-directional entity, can be modulated by exercise and ambient temperature, influencing susceptibility to infections, illnesses, and pathological conditions [1], [2]. Acute bouts of exercise modulate immune functions by increasing leukocyte count and subsets with a subsequent release of immuno-mediators, including pro- and anti-inflammatory cytokines, interleukins (IL) 6, -1ra, -8, -10, -12, growth factors and other signaling proteins [3], [4]. The intensity of exercise also effects immune responses with regular low- and moderate-intensity exercise generally promoting positive immune outcomes [5], [6], [7].

Cold exposure is generally used as a broad concept that: *i*) includes multiple levels of physiological effects depending of the degree of cooling (vasoconstriction, hormonal secretion, bioenergetics, muscular control, etc.) and *ii*) has high inter-individual response variability [8], [9]. Previous investigations on the effects of cold exposure on the immune system showed minimal or no alterations in immunological functions [10], [11]. Nonetheless, lower cytokine production and lymphocyte proliferation has been observed with decreased core temperature [12] and normothermic subjects exposed to cold air [13]. Rhind et al. [14] determined that cold exposure may modulate cytokine production by selectively upregulating IL-1 and IL-6 while downregulating IL-1β and TNF-α, associating these results with cold-induced catecholamine secretion. Nonetheless, LaVoy et al. [15] reviewed 10 studies examining the effects of exercising in the cold on various immune outcomes and found no clear tendencies across studies for either an increase or a decrease in immune functions.

Shivering thermogenesis is a common thermoregulatory stress response that can occur early during cold exposure constituting of involuntary and repetitive muscle contractions to maintain thermal homeostasis by producing heat. The contribution of core cooling to shivering activity is ∼67–80%, whereas skin and peripheral tissue cooling account for the remaining portion [16], [17]. Shivering in the cold increases cortisol and norepinephrine concentrations, known to modulate immune responses [3], at rest [18], [19] and during exercise in the cold [20]. The examination of various cold thermal challenges altering endocrine responses, including shivering thermogenesis, could consequently help explain some immune differences from previous reports.

The variability in environmental conditions between studies [21], [22] complicates the comparison of results as temperatures nearing 5–10°C provided little to no thermal stress due to the increased metabolic heat production from exercise [20]. Additionally, some studies have evaluated immune differences between a cool and a hot environment, neglecting the comparison to a thermoneutral setting where immunity should be unchallenged and represent a control group comparison [21], [22]. It is therefore important to control laboratory environmental settings as its influences on endocrine responses are closely associated with exercise intensity and environmental conditions, and are responsible for immune changes including leukocyte and cytokine responses [14], [23], [24].

Studies examining immuno-endocrine modulations have investigated the implications of the sympathetic nervous system (SNS) (norepinephrine [NE] and epinephrine [Epi]), and the hypothalamic-pituitary-adrenal axis (HPA) (cortisol [COR] and adrenocorticotropic hormone [ACTH]) to explain the variance in cytokine and leukocyte responses [3], [14], [25]. The control mechanisms of these hormones on the immune system have been well studied [3], [14], [26], [27], [28], [29]. Nonetheless, immune responses are further modulated by the hypothalamic-pituitary-thyroid axis (HPT) and the hypothalamic-pituitary-gonadal axis (HPG) [30], [31]. The limited work performed on immune function during exercise in the cold have examined leukocytes and a limited number of cytokines simultaneously with NE, Epi and COR secretion only [3], [11], [14], [21]. To obtain a comprehensive outlook of the immune system under cold challenges, investigating the effects of other endocrine axes in addition to a broader inventory of immunity-modulating cytokines is required.

The purposes of the present study were: *i*) to examine the effects of low- and moderate-intensity exercise in a thermoneutral and cold environment, with and without the presence of shivering on leukocytes and leukocyte subsets, and hormone and cytokine responses; and *ii*) to investigate the effects of a thermoneutral and cold environment, with and without the presence of shivering on the relationships between immunological (leukocytes and cytokines) and endocrine (hormones from the HPA, SNS, HPT and HPG axes) parameters. We hypothesized that immuno-endocrine responses would be diminished in colder conditions (from a cold condition as well as with shivering and/or at lower exercise intensity), influencing serum hormonal concentrations and subsequently leukocytes and cytokines concentrations. We also hypothesized that all endocrine axes will influence the immune responses across environmental conditions.

## Methods

Nine moderately active and not cold acclimatized male participants volunteered for the study. They each provided written informed consent and were screened with a PAR-Q and for cardiovascular and respiratory conditions that could be aggravated by cold air exposure or exercise. None of the participants were on prescribed medications. Mean ±SD characteristics of the participants were determined during a familiarization trial and were: age 24±2 yrs; height 181.4±10.2 cm; body mass 83.1±9.7 kg; body fat 19.1±4.8%; body surface area 2.04±0.18 m^2^; and peak oxygen consumption 52.9±5.2 ml·kg^−1^·min^−1^. The study was performed according to the declaration of Helsinki and was approved by the Ethical Committee of the Central Finland Health Care District.

### Experimental protocol

Each participant took part in 6 experimental sessions, wearing shorts and t-shirt (clothing equivalent of ∼0.2 to 0.3 clo), separated by at least 72 h and at the same time of day to control for circadian rhythms. They arrived at the laboratory between 0700 and 0800 h, preceded by a 24-h period without alcohol, caffeine, tobacco and vigorous exercise.

Instrumentation of the participants took approximately 45 min and was performed in a climatic chamber set at 25.0±0.2°C, 40% relative humidity (RH) and 0.2 m·s^−1^ wind speed. Following instrumentation, subjects sat for a 15 min baseline period in the same chamber. Thereafter, the participants moved to the adjacent experimental chamber set at either 22.0 or 0.0±0.2°C, 40% RH and 0.2 m·s^−1^ wind speed. Then, they either immediately started exercising, or remained seated (in 0°C only) until oxygen consumption from shivering thermogenesis activity averaged 15% of peak oxygen consumption (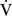
O_2peak_), equivalent to low-intensity shivering. Pre-exercise cooling time ranged from 40 to 120 min depending of the participant. This protocol has previously demonstrated that combined exercise and shivering can co-exist in the early stages of exercise (i.e. 15–30 min) and influence endocrine responses in a cold environment [20]. Low-intensity shivering was chosen as moderate and high-intensity shivering may impede neuromuscular function and prevent task completion such that combined exercise and shivering activity would typically only occur during low-intensity shivering. Exercise was comprised of 60 min of treadmill exercise at a grade of 1.0% (Tunturi T40, Accell Group, Heerenveen, The Netherlands).

Experimental sessions followed a balanced factorial design and involved the following six conditions: 1) Exercise at 50% 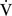
O_2peak_ (low-intensity exercise) in a thermoneutral (22°C, NT) condition (LOW NT); 2) Exercise at 50% 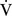
O_2 peak_ in a cold (0°C, COLD) condition (LOW COLD); 3) Exercise at 50% 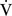
O_2 peak_ in a cold condition following pre-exercise cooling leading to low-intensity shivering (0°C, SHIV) (LOW SHIV); 4) Exercise at 70% 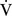
O_2 peak_ (moderate-intensity exercise) in a thermoneutral condition (MOD NT); and 5) Exercise at 70% 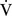
O_2peak_ in a cold condition (MOD COLD); and 6) Exercise at 70% 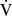
O_2peak_ in a cold condition following low-intensity shivering (MOD SHIV). Low-intensity exercise was performed via walking while moderate-intensity exercise was done via running. Treadmill speed for each subject corresponding to the protocol intensities was determined individually during the familiarization trial and manually adjusted if needed during experimental trials to ensure that relative %
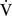
O_2peak_ remained constant throughout the full 60 min of exercise. This protocol was deemed to mimic natural pacing behavior as opposed to following a specific workload regardless of environmental conditions or physiological demands [20], [32].

### Instrumentation and measurements

The participants were instrumented while standing in the thermoneutral instrumentation chamber. Together with the 15 min baseline period, this ensured that subjects were in a physiologically thermoneutral state prior to testing. Rectal temperature, representing core temperature (T_core_), was measured using a rectal thermistor (YSI 401, Yellow Springs Instruments, USA) inserted 10 cm beyond the anal sphincter. Skin temperature was measured from 6 sites (face, chest, forearm, hand, thigh, and back) using thermistors (NTC DC95, Digi-Key, USA). Both core and skin temperature data were recorded by a portable data logger (SmartReader Plus 8, ACR Systems Inc., Surrey, Canada). Weighted mean skin temperature (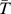
_sk_) was subsequently calculated using the weighted average of the 6 sites [33]:


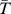
_sk_ = 0.14 (T_face_)+0.19 (T_chest_)+0.11 (T_forearm_)+0.05 (T_hand_)+0.32 (T_thigh_)+0.19 (T_back_)

Heart rate (HR) was continuously monitored and recorded with a heart rate monitor (T6, Suunto, Vantaa, Finland). Oxygen consumption (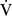
O_2_) and respiratory exchange ratio (RER) were measured using an open circuit ergospirometer with a gas mixing chamber (Medikro 919, Kuopio, Finland). A one-way Hans-Rudolph valve, connected to a breathing tube, was used in all trials to collect expired gases. Gas collection and mixing was done outside of the climatic chamber at thermoneutral temperature (25°C).

### Blood sampling and analyses

An OCRILON polyurethane catheter (Optivia I.V 18G, Jelco, Smith's Medical, Ashford, UK), positioned in the antecubital vein before the start of the experiment and maintained throughout exercise, was used to collect blood samples in 3.5-ml vacuum-sealed serum tubes with silicon coating (BD Vacutainer SST tubes, BD, New Jersey, USA) and in 3-ml K_2_EDTA whole blood tubes (BD Vacutainer Plus Plastic K_2_EDTA tubes, BD, New Jersey, USA). Catheters were maintained using adhesive hypoallergenic, water-resistant tape (3M Transpore Surgical Tape, 3M Health Care, London, Canada). The blood samples in serum tubes were given 30 min to coagulate as recommended by the manufacturer. Centrifugation was then performed at 3500 rpm for 10 min (4100 g) followed by isolation of plasma and serum samples in Eppendorf tubes and frozen at -80°C for future analysis.

Serum cortisol (COR), adrenocorticotropic hormone (ACTH), total testosterone (TES_tot_), sex-hormone-binding globulin (SHBG), thyroid-stimulating hormone (TSH), free thyroxine (T_4free_), triiodothyronine (T_3_) and free triiodothyronine (T_3free_) were analyzed by Immulite 1000 (DPC Diagnostics Corporation, Los Angeles, USA) using respective commercial luminoimmunoassay kits (Ortho Clinical Diagnostics, Amersham, UK) and Immulite 2000 with chemiluminescent immunometric assay kits (Siemens Healthcare, Diagnostics Products Ltd, Llanberis, UK). The sensitivity and intra-assay coefficients of variance for these assays were 5.5 nmol·L^−1^ and <7.4%, 5.0 ng·L^−1^and <3.8%, 0.5 nmol·L^−1^ and <8.4%, 0.2 nmol·L^−1^ and <7.3%, 0.004mU·L^−1^ and <7.9%, 1.67 pmol·L^−1^ and <5.5%, 0.54 nmol·L^−1^ and <7.5%, and finally, 1.0 pg·ml^−1^ and <5.7%, for COR, ACTH, TES_tot_, SHBG, TSH, FT4, F3 and FT3. Bioavailable testosterone (TES_bio_) was estimated using the equation of Morris et al. (2004): lnTES_bio_ = −0.266+(0.955*lnTES) – (0.228*lnSHBG) (ln  =  natural log, units are in nmol/L). Calculated TES_bio_ and measured TES_bio_ have previously demonstrated to be highly correlated (r = 0.90) with each other [34].

Plasma catecholamine concentrations (epinephrine [Epi] and norepinephrine [NE]) were analyzed via a commercial ELISA kit (DRG Instruments GmbH, Germany). Coefficients of variance for intra-assay precision were 15.0% for Epi and 16.1% for NE at 2.5 ng·ml^−1^and 24.4 ng·ml^−1^ levels, respectively. Plasma samples for leukocyte count and subsets (lymphocytes, monocytes, and granulocytes) (x10^3^ cells·µL^−1^) were mixed immediately after collection on a roller for 3 min as recommended by the manufacturer. Samples were then analyzed in triplicate following the Coulter principle of counting and sizing by a hematological analyzer (Coulter A^c^·T diff, Beckman Coulter Finland Oy, Vantaa, Finland).

Plasma cytokines were measured with the Bio-Plex 200 system based on Luminex xMAP technology (BioRad Laboratories Inc., CA, USA) and Bio-Plex Pro Human preselected Cytokine 27-plex Assay (CAT# M50-0KAF0Y, BioRad Laboratories Inc., CA, USA). The cytokines assessed included interleukins 1 receptor antagonist (IL-1), 1 receptor beta (IL-1β), 2 (IL-2), 4 (IL-4), 5 (IL-5), 6 (IL-6), 7 (IL-7), 8 (IL-8), 9 (IL-9), 10 (IL-10), 12 (IL-12), 15 (IL-15), 17 (IL-17), eotaxin, basic fibroblast growth factor (FGF2), granulocyte colony-stimulating factor (G-CFS), interferon gamma (IFN-γ), interferon gamma-induced protein 10 (IP-10), platelet-derived growth factor (PDGF), monocyte chemoattractant protein-1 (MCP-1), macrophage inflammatory protein 1 beta (MIP-1β), rantes and vascular endothelial growth factor (VEGF). The assay was performed according to manufacturer's instructions as previously described [35], [36]. The plasma samples were thawed on ice and centrifuged at 13 000 G for 10 min in 4°C before the assay. For the assay, the samples were diluted 1∶2 in assay buffer. The washing of the plate was done with BioTek ELx405 plate washer (BioTek Instruments, Inc., VT, USA) to minimize variation. The results were calculated with Bio-Plex Manager Software 6.0 with five parameter logistical equation.

### Statistical analyses

Generalized estimating equations (GEE) models were constructed for a factorial design of environment (levels: NT (thermoneutral), COLD (cold), and SHIV (cold and shivering), exercise intensity (levels: LOW (low intensity) and MOD (moderate intensity), and time (levels: baseline (BL) and end of exercise (EEx)) to determine the significances of changes in HR, T_core_, 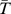
_sk_, leukocyte count and subsets, hormones and cytokines. The GEE models permit the estimation of an unstructured within-subjects correlation matrix that adjusts for dependencies arising from repeated-measurements (times) and the nesting of the environment and intensity levels within measurement time-points. Post-hoc analyses were conducted using independent Bonferroni test when appropriate. Heart rate, core and skin temperature at EEx were calculated from the average of the last 15 min of exercise. Pearson's product-moment correlation coefficients were calculated over time and exercise intensity to assess potential relationships between cardiovascular, thermoregulatory and endocrine responses with cytokine responses, separately in the three environmental conditions. To examine the combined acute effects of cytokine changes from exercise and cold exposure, growth factors, anti-inflammatory and pro-inflammatory cytokines values were divided, converted into a z-score and then computed into an index to be subsequently analyzed via a one-way RM ANOVA with the factor of environment (levels: NT, COLD, and SHIV). The results are reported as mean ± SD by using *p*<0.05 to identify statistical differences. All analyses were performed using the statistical software package SPSS 19 for Windows (IBM, Armonk, NY, USA).

## Results

### Thermoregulatory and cardiovascular measures

Thermoregulatory parameters and heart rate values are presented in Table 1. Heart rate was greater in NT *vs.* COLD in MOD at EEx (*p*<0.05). For core temperature, the increase in heat production from exercise was lower in SHIV by 0.3°C compared to NT and COLD within the LOW condition (*p*<0.05). In MOD, the increase in core temperature was greater in NT compared to COLD and SHIV by 0.4°C. The decrease in skin temperature from cold exposure in COLD and SHIV conditions was lower in SHIV compared to COLD by 2.0°C in LOW only.

**Table 1:** Mean ± SD values for heart rate (HR), core temperature (T_core_) and mean skin temperature (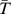
_sk_) following 60 min of low and moderate exercise in a thermoneutral (NT), cold (COLD) and cold environment with shivering (SHIV) at baseline (BL) and at end of exercise (EEx).

*CARDIOVASCULAR AND*			*LOW*			*MOD*	
*THERMOREGULATORY VARIABLES*		*NT*	*COLD*	*SHIV*	*NT*	*COLD*	*SHIV*
HR (beats·min^−1^)	BL	67±14	67±16	69±9	67±13	68±12	71±15
	EEx	140±10*	128±13*	130±18*	176±13^B,^ *	161±16*	165±14*
T_core_(°C)	BL	36.9±0.2	37.0±0.2	37.1±0.2	37.0±0.1	37.0±0.1	37.0±0.1
	EEx	37.8±0.2*	37.8±0.4*	37.5±0.6^B,C,^ *	38.8±0.2^A,B,^ *	38.4±0.2*	38.4±0.3*
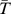 _sk_ (°C)	BL	33.2±0.6	32.6±0.7	32.5±0.4	32.7±1.0	32.6±0.6	32.7±0.8
	EEx	31.7±1.2^A,B^	21.7±2.9^A,^ *	19.7±1.3*	32.4±1.9^A,B^	23.9±1.4*	23.0±2.5*

*, Significant difference between pre and post (*p*<0.05).

A, Significantly different from SHIV (*p*<0.05).

B, Significantly different from COLD (*p*<0.05).

C, Significantly different from NT (*p*<0.05).

### Endocrine changes

Table 2 presents changes of measured hormones in all conditions. At EEx, total testosterone was greater in the NT condition compared to SHIV and COLD in both LOW and MOD. For bioavailable testosterone, however, NT was only greater than SHIV. While there was a tendency for total and bioavailable testosterone to decrease in the SHIV condition, statistical significance was not achieved. The response of insulin-like growth factor 1 demonstrated an increase at EEx in all conditions in MOD only with values greater in NT (40.66±12.54 nmol·L^−1^) compared to COLD (35.51±10.81nmol·L^−1^). The adrenocorticotropic hormone and cortisol responses exhibited no changes from exercise in LOW. In MOD, however, the adrenocorticotropic hormone and cortisol values in NT increased from BL and were greater compared to the COLD and SHIV conditions. Regarding catecholamines, both norepinephrine and epinephrine demonstrated increases at EEx. Additionally, norepinephrine concentration in the COLD condition was lower compared to SHIV and NT across exercise intensities. Lastly, thyroid hormones all thyroid hormones concentrations demonstrated increases at EEx. Nonetheless, only free triiodothyronine concentration showed a condition difference as the SHIV condition demonstrated lower concentration (2.69±0.47 pg·ml^−1^) compared to NT (2.30±0.65 pg·ml^−1^).

**Table 2:** Mean ± SD concentrations for total testosterone (TES_tot_), bioavailable testosterone (TES_bio_), sex hormone-binding globulin (SHBG), insulin-like growth factor 1 (IGF-1), adenocorticotropin hormone (ACTH), cortisol (COR), norepinephrine (NE), epinephrine (Epi), thyroid-stimulating hormone (TSH), triiodothyronine (T3), free triiodothyronine (T_3free_), and free thyroxine (T_4free_) following 60 min of low and moderate exercise in a thermoneutral (NT), cold (COLD) and cold environment with shivering (SHIV) at baseline (BL) and at end of exercise (EEx).

*HORMONES*			*LOW*			*MOD*	
		*NT*	*COLD*	*SHIV*	*NT*	*COLD*	*SHIV*
TES_tot_ (nmol·L^−1^)	BL	16.17±3.99	16.85±3.76	15.63±4.44	14.67±4.02	14.71±4.01	16.28±3.69
	EEx	19.77±7.53^A,B^	15.93±4.62	15.10±4.41	19.97±7.76^A,B,^ *	15.73±5.91	16.10±6.45
TES_bio_(nmol·L^−1^)	BL	0.71±0.17	0.74±0.15	0.68±0.18	0.65±0.23	0.66±0.17	0.72±0.14
	EEx	0.76±0.23^A^	0.68±0.19	0.66±0.17	0.77±0.25^A^	0.67±0.20	0.65±0.31
SHBG (nmol·L^−1^)	BL	39.84±12.66	36.61±11.49	40.24±7.54	38.46±12.98	41.53±9.36	41.89±11.93
	EEx	42.94±13.56	39.15±10.89	46.37±8.32^B,^ *	40.86±12.10	43.267±10.83	42.04±11.17‡
IGF-1 (nmol·L^−1^)	BL	31.34±8.82	31.23±11.07	30.22±6.14	35.23±10.53	29.59±11.04	32.50±8.46
	EEx	35.16±9.57	35.42±9.24	32.51±6.38	40.66±12.54^B,^ *	35.51±10.81*	37.59±9.58*
ACTH (nmol·L^−1^)	BL	16.80±6.99	21.61±8.29	22.39±18.19	22.30±13.31	17.07±9.25	17.78±8.88
	EEx	22.2±7.63	20.12±12.46	17.04±9.74	45.50±19.05^A,B,^ *	21.99±5.13	18.73±6.94
COR (nmol·L^−1^)	BL	413±116	468±150	444±166	493±149	433±109	459±142
	EEx	413±110	453±204	457±143	593±133^A,B,^ *	444±104	464±162
NE (ng·ml^−1^)	BL	0.37±0.13	0.66±0.54	0.54±0.23	0.45±0.15	0.40±0.26	0.66±0.89
	EEx	1.18±0.48	1.07±0.66^E,^ F	1.76±1.30†	2.46±1.30*	1.11±0.60	1.44±0.80†
Epi (ng·ml^−1^)	BL	0.05±0.02	0.04±0.02	0.06±0.03	0.04±0.01	0.05±0.02	0.05±0.03
	EEx	0.08±0.06	0.08±0.05	0.09±0.04†	0.11±0.05	0.09±0.07	0.09±0.06†
TSH (mU·L^−1^)	BL	1.46±0.73	1.51±0.96	1.47±0.58	1.45±0.47	1.44±0.81	1.43±0.65
	EEx	1.63±0.88	1.48±1.06	1.57±0.79	1.76±0.69	1.82±1.43	1.54±0.82†
T_3_ (nmol·L^−1^)	BL	1.28±0.36	1.33±0.40	1.33±0.29	1.30±0.25	1.26±0.29	1.38±0.35
	EEx	1.35±0.34	1.41±0.37	1.47±0.23	1.38±0.33	1.32±0.30	1.35±0.33‡
T_3free_(pg·ml^−1^)	BL	2.18±0.63	2.32±0.65	2.40±0.68	2.09±0.52	2.02±0.46	2.32±0.71
	EEx	2.30±0.65	2.55±0.43	2.69±0.47^C,^ *	2.31±0.55	2.20±0.55	2.27±0.62‡
T_4free_ (pmol·L^−1^)	BL	15.87±2.83	14.47±2.12	14.35±1.93	15.29±2.17	14.31±1.97	15.19±2.51
	EEx	15.90±3.07	15.02±2.37	15.00±1.94	15.71±2.41	15.07±1.45	16.08±2.03‡

*, Significant difference between pre and post (*p*<0.05).

† , Significant difference between pre and post across environmental conditions.

‡ difference between pre and post across environmental and exercise conditions.

A, Significantly different from SHIV (*p*<0.05).

B, Significantly different from COLD (*p*<0.05).

C, Significantly different from NT (*p*<0.05).

D, Significantly different from COLD across exercise intensities (*p*<0.05).

E, Significantly different from NT across exercise intensities (*p*<0.05).

F , Significantly different from SHIV across exercise intensities (*p*<0.05).

### Leukocytes

**Figure 1:**
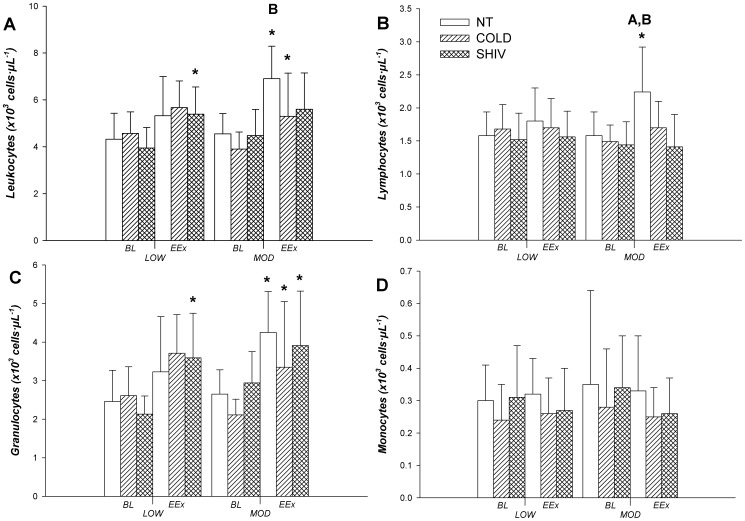
Changes in leukocytes (A), lymphocytes (B), granulocytes (C), and monocytes (D) between baseline (BL) and end of 60 min of exercise (EEx) (N = 9) (Mean ±SD). *Significantly different between BL and EEx (*p*<0.05). ^A^ Significantly different from SHIV (*p*<0.05). ^B^ Significantly different from COLD (*p*<0.05).

Total leukocytes increased at EEx compared to BL in LOW SHIV. In MOD, an increase in leukocytes was seen in NT and COLD (Fig. 1A) with NT being greater than COLD. Lymphocytes increased over time in NT *vs.* COLD and SHIV in MOD (Fig. 1B). Granulocytes demonstrated increases at EEx in all conditions in MOD but only in SHIV in LOW (Fig. 1C). No differences between environmental conditions were observed in granulocytes or monocytes (Fig. 1D).

Correlation coefficients for leukocytes, lymphocytes, granulocytes and monocytes with HR, core temperature, skin temperature, and hormones in all environmental conditions are presented in Tables S1, S2, and S3. Importantly, norepinephrine was associated to leukocytes in the NT condition only (r = 0.544) while cortisol was associated to leukocytes in NT and SHIV but not COLD (r = 0.550). The COLD condition presented to lowest amount of associations between parameters. Finally, free triiodothyronine in the SHIV condition was negatively associated with all interleukins and other cytokines.

### Cytokines

**Figure 2:**
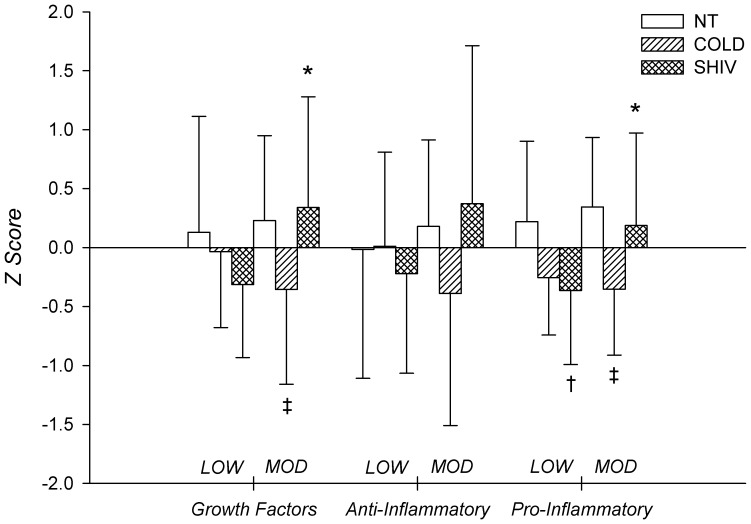
Z scores for growth factors, anti-inflammatory and pro-inflammatory cytokines in NT, COLD and SHIV within LOW and MOD. *Significantly different between LOW and MOD (*p*<0.05). ^†^Significantly different from NT (*p*<0.05). ^‡^Significantly different from NT and SHIV (*p*<0.05).

Changes in pro-inflammatory, anti-inflammatory as well as growth factor cytokines are presented in Tables 3, 4, and 5. Correlation coefficients between cytokines and HR, core temperature, skin temperature, and hormones in all environmental conditions are presented in Tables S1, S2, and S3. When weighted and indexed through a z score (Fig. 2), the immune response for growth factors, indicated a greater response in MOD SHIV compared to LOW SHIV. Furthermore, within MOD, cytokine responses in COLD was significantly lower than both NT and SHIV. The pro-inflammatory cytokines response also indicated a greater response of SHIV in MOD compared to LOW. Finally, pro-inflammatory cytokines in SHIV were lower than NT in LOW, and lower in COLD compared to SHIV and NT in MOD.

**Table 3:** Mean ± SD concentrations (ng·ml^−1^) of pro-inflammatory cytokines following 60 min of low and moderate exercise in a thermoneutral (NT), cold (COLD) and cold environment with shivering (SHIV) at baseline (BL) and at end of exercise (EEx).

*PRO-INFLAMMATORY CYTOKINES*			*LOW*			*MOD*	
		*NT*	*COLD*	*SHIV*	*NT*	*COLD*	*SHIV*
IL-1β	BL	1.55±0.97	1.92±0.82	1.68±0.86	1.63±0.93	1.67±0.71	1.83±1.12
	EEx	3.30±1.41	2.47±0.85^E,^ F	2.26±1.09†	3.13±0.92	2.11±1.06	3.53±1.44 * † ‡
IL-2	BL	3.33±2.33	4.79±1.97	4.09±2.29	4.50±2.34	3.37±2.00	4.44±2.81
	EEx	8.04±3.28^D,^ *	6.39±2.21	5.39±2.51	8.30±3.28*	5.63±2.82	8.36±3.39* ‡
IL-5	BL	3.64±1.27	3.79±1.11	3.49±0.97	3.96±0.87	3.29±1.10	3.77±1.82
	EEx	5.34±1.38	5.24±1.55	4.97±1.48	5.32±1.24	4.74±1.57	5.47±1.94‡
IL-6	BL	9.32±3.06	10.58±3.26	8.98±3.43	9.90±2.68	8.82±2.90	10.20±4.44
	EEx	12.28±3.49*	12.75±2.51	11.94±4.04*	13.51±3.22*	11.53±4.03*	13.79±3.89* ‡
IL-7	BL	16.63±6.25	19.42±6.12	16.11±6.89	17.86±8.59	16.36±7.35	16.16±6.09
	EEx	22.40±7.67^D,^ F	16.62±5.75	14.43±3.56	22.89±8.9	14.95±5.57	22.61±11.01‡
IL-8	BL	7.12±4.81	7.90±5.07	6.69±4.44	7.60±5.44	6.11±4.86	6.97±5.12
	EEx	8.04±4.20	8.28±5.11	7.43±5.33	8.61±5.02	7.77±5.38	8.70±4.95†
IL-9	BL	23.12±22.52	27.53±21.78	23.27±16.06	19.99±13.04	17.56±16.22	28.28±19.54
	EEx	34.53±24.69	30.19±16.36	26.10±18.46	34.38±23.93*	25.65±13.89	35.55±22.23† ‡
IL-12	BL	33.47±18.38	37.38±19.47	34.08±18.83	39.44±20.93	29.07±19.83	33.64±22.73
	EEx	26.76±13.24^D^	41.56±14.75	40.50±17.48	26.89±12.45	36.85±22.26	28.86±14.31
IL-15	BL	5.83±2.96	6.74±2.93	5.34±3.07	5.78±2.81	5.08±3.00	5.75±3.79
	EEx	7.54±2.56	7.23±1.94	6.60±2.86	7.79±2.55*	6.65±2.76	7.85±3.62‡
IL-17	BL	67.10±64.19	71.04±61.28	62.63±67.06	72.64±62.29	54.43±63.61	60.47±65.17
	EEx	83.68±67.28	82.78±54.85	70.19±66.66	93.65±78.92^B^	70.21±62.39	91.16±69.63* † ‡
IFN-γ	BL	110.26±38.55	118.49±29.05	110.34±46.30	116.46±41.21	104.68±48.91	115.81±53.89
	EEx	173.87±54.16^A,B,D,^ F ^,^ *	132.36±35.01	124.74±43.49	175.85±55.46*	125.37±43.84	172.50±64.22^B,^ * ‡
Rantes	BL	818±201	847±160	912±122	895±135	932±109	897±141
	EEx	1041±209^A,B,D,^ *	740±160	785±75*	1078±130*	832±160^A,C^	1029±302*
Eotaxin	BL	83.74±19.36	87.78±12.08	76.16±16.20	78.69±15.98	77.74±8.68	80.05±12.82
	EEx	99.25±20.20^A,B,D,^ F	75.75±11.41	77.10±12.21	96.70±17.35	78.64±11.50	86.09±19.70‡
IP-10	BL	854±348	709±233	674±229	861±720	656±172	866±550
	EEx	887±294^D,^ F	605±219	593±171	1150±798^A,B^	656±193	645±223
MIP-1β	BL	31.27±8.24	32.81±6.48	30.97±7.58	33.45±7.92	27.98±7.42	34.17±12.81
	EEx	36.48±9.19^A,B,D,^ F	29.59±6.28	27.56±7.79^D^	38.65±8.59^B^	29.24±7.38	39.01±11.92^B^
MCP-1	BL	12.71±4.43	12.37±3.40	12.29±3.68	11.82±3.39	12.09±3.11	13.90±6.31
	EEx	14.94±4.33^A,B, D,^ F	9.33±2.24	9.43±1.92	16.49±5.07^B,^ *	11.33±3.49	15.79±7.27^B,^

*, Significant difference between BL and EEx (*p*<0.05).

† , Significant difference between BL and EEx across environmental conditions.

‡ difference between BL and EEx across environmental and exercise conditions

A, Significantly different from SHIV (*p*<0.05).

B, Significantly different from COLD (*p*<0.05).

C, Significantly different from NT (*p*<0.05).

D, Significantly different from COLD across exercise intensities (*p*<0.05).

E, Significantly different from NT across exercise intensities (*p*<0.05).

F , Significantly different from SHIV across exercise intensities (*p*<0.05).

**Table 4:** Mean ± SD concentrations (ng·ml^−1^) of anti-inflammatory cytokines following 60 min of low and moderate exercise in a thermoneutral (NT), cold (COLD) and cold environment with shivering (SHIV) at baseline (BL) and at end of exercise (EEx).

*ANTI-INFLAMMATORY CYTOKINES*			*LOW*			*MOD*	
		*NT*	*COLD*	*SHIV*	*NT*	*COLD*	*SHIV*
IL-1ra	BL	300±119	298±80	262±83	287±74	242±69	287±119
	EEx	437±167*	401±113	386±109	442±119*	347±144	471±154* ‡
IL-4	BL	2.73±0.76	2.97±0.83	2.71±0.85	2.93±0.81	2.49±0.60	2.84±1.11
	EEx	3.45±1.13	3.74±0.80	3.43±1.01	3.49±0.56	3.37±1.15	3.74±1.38‡
IL-10	BL	2.42±1.16	2.45±0.93	2.47±0.57	2.54±0.86	1.44±0.98	3.34±1.69
	EEx	3.13±1.29	3.34±1.21	2.98±1.34	4.31±1.82^B,^ *	3.05±1.80*	4.47±2.22^B,^ * ‡

*, Significant difference between pre and post (*p*<0.05).

† , Significant difference between pre and post across environmental conditions.

‡ difference between pre and post across environmental and exercise conditions.

A, Significantly different from SHIV (*p*<0.05).

B, Significantly different from COLD (*p*<0.05).

C, Significantly different from NT (*p*<0.05).

D, Significantly different from COLD across exercise intensities (*p*<0.05).

E, Significantly different from NT across exercise intensities (*p*<0.05). F, Significantly different from SHIV across exercise intensities (*p*<0.05).

**Table 5:** Mean ± SD concentrations (ng·ml^−1^) of growth factors following 60 min of low and moderate exercise in a thermoneutral (NT), cold (COLD) and cold environment with shivering (SHIV) at baseline (BL) and at end of exercise (EEx).

*GROWTH MEDIATES AND COLONY-STIMULATING FACTOR*			*LOW*			*MOD*	
		*NT*	*COLD*	*SHIV*	*NT*	*COLD*	*SHIV*
VEGF	BL	27.02±12.76	31.84±16.95	30.11±18.20	33.61±15.50	23.96±15.90	26.84±19.24
	EEx	44.23±21.41^D,^ *	38.70±17.20	34.35±17.56	50.52±24.91^B,^ *	34.95±20.58*	51.57±22.33^B,^ * ‡
FGF	BL	34.66±30.63	39.72±33.71	32.95±32.95	37.18±30.55	30.58±35.52	32.33±35.96
	EEx	47.22±32.62	40.86±26.68	33.43±29.21	48.67±35.23	36.48±31.62	49.26±33.09† ‡
PDGF	BL	478±248	409±251	390±198	482±271	294±126	417±269
	EEx	1398±922^A,B,^ *	775±366^D,E^	680±199	1353±563^B,^ *	709±292	1567±1012^B,^ * ‡
G-CSF	BL	31.56±8.66	34.28±5.06	28.93±9.85	32.48±5.85	27.89±7.09	32.03±8.61
	EEx	38.13±11.29	45.22±8.41^D,E^	40.09±10.06	39.21±9.17^B^	36.77±11.85	39.87±10.78‡

*, Significant difference between pre and post (*p*<0.05).

† , Significant difference between pre and post across environmental conditions.

‡ difference between pre and post across environmental and exercise conditions.

A, Significantly different from SHIV (*p*<0.05).

B, Significantly different from COLD (*p*<0.05).

C, Significantly different from NT (*p*<0.05).

D, Significantly different from COLD across exercise intensities (*p*<0.05).

E, Significantly different from NT across exercise intensities (*p*<0.05).

F , Significantly different from SHIV across exercise intensities (*p*<0.05).

## Discussion

The present experimental protocol elicited a change in all endocrine axes and some immunological measures. The main findings of this study were that: *i*) exercising in a thermoneutral environment elicited a stronger response in a large array of endocrine and immunological measures suggesting an immuno-depressive effect from the cold on exercise-related immuno-endocrine responses; and, *ii*) the presence of shivering prior to and during the early stages of exercise in the cold may induce an immuno-stimulatory effect during moderate exercise intensities. The first finding was determined based on the greater responses of leukocytes, lymphocytes, total testosterone, bioavailable testosterone, adrenocorticotropic hormone, cortisol, insulin-like growth factor 1, NE, IL-2, IL-7, IL-12, IL-17, IFN-γ, Rantes, Eotaxin, IP-10, MIP-1β and MCP-1 in a thermoneutral compared to a cold environment. The second finding was determined by increases in norepinephrine, free triiodothyronine, some growth factors and pro-inflammatory cytokines in the SHIV condition compared to COLD. When cytokines were indexed on a standardized z score, a similar response was seen across conditions for growth factors, as well as anti- and pro-inflammatory cytokines. Specifically, growth factors and pro-inflammatory cytokines were elevated in MOD SHIV compared to LOW SHIV and were greater than COLD at both intensities. Moreover, associations between endocrine and immunological measures provided diverse outcomes between environmental conditions suggesting weaker relationships in the cold between endocrine and immunological parameters.

Other reports examining the immunomodulatory effects of temperature used cold environmental protocols of 8°C and above [37], [38], [39], suggesting a limited intensity of the cold stimulus compared to the present study. The present data, collected in a thermoneutral (22°C) *vs.* cold (0°C) environments, confirms previous findings indicating upregulated endocrine changes from increased thermal stress (i.e. elevated core and skin temperature) as an indirect pathway in stimulating secretion of norepinephrine, cortisol and other hormones, thereby influencing and increasing immune responses [14], [23], [24].

The SHIV condition, nonetheless, responded in a different manner, irrespective of core temperature changes, suggesting another mechanism. The additional cold stress prior to exercise may have been sufficient to induce the increase in free triiodothyronine, a hormones known to affect whole-body metabolism and heat production [19], leading to its implication on cytokine production, mainly interleukins. Acute cold exposure generally does not induce a noticeable increase in thyroid hormones as thermal homeostasis may not be compromised or since the hypothalamic-pituitary-thyroid axis response to stress is much slower compared to the sympathetic nervous system or sympatho-adrenal medullary axis. Additional cooling, as in our case with shivering, did increase free triiodothyronine, modulating cytokine responses. Although in lower circulating quantity than free thyroxine, free triiodothyronine is a more potent hormone on targeted cells. Limited work has been done to demonstrate an interaction between the hypothalamic-pituitary-thyroid axis and the immune system. Klecha et al. [40] determined a regulatory role of the thyroid-stimulating hormone, triiodothyronine, and thyroxine on cytokine production in lymphocytes in mice and attributed the interaction via protein kinase C enzymatic pathway between both systems. While the present study does not offer a clear mechanistic approach to the results, the implication of the hypothalamic-pituitary-thyroid axis in regulating cytokine production in the SHIV condition seems likely. The greater norepinephrine response seen in SHIV compared to COLD was likely a strong factor in increasing immune responses. Norepinephrine has previously been established as an important hormone in the bi-directional immuno-endocrine system targeting β-adrenoreceptors on immune cells [25].

### Endocrine functions

Endocrine modulations were limited in the LOW condition since the increase in core temperature was restricted to 0.9°C in NT, 0.8°C in COLD and 0.4°C in SHIV, and physical demand was limited to 50% of VO_2peak_. Although Rhind et al. [24] suggested that an increase of over 0.5°C in core temperature represented a thermal threshold for stress hormone release and subsequent cytokine production, only total testosterone, bioavailable testosterone and norepinephrine were modulated in LOW. Increases in testosterone in a thermoneutral environment, associated with some endurance exercise protocols [41], [42], have been reported, but the underlying mechanisms for its absence in the other conditions remains to be determined. Literature suggests limited to no change in serum testosterone concentration from acute cold air exposure [18]. Since luteinizing hormone, a stimulating hormone produced in the anterior pituitary gland, does not seem to change under cold exposure either [18], higher order mechanisms from the hypothalamus and the gonadotropin-releasing hormone could explain our results. Concerning the greater norepinephrine concentrations observed in NT and SHIV compared to COLD, this response can be explained by the greater heat stress generated in NT [43] and the additional cooling effects in SHIV on the sympathetic nervous system prior to exercise [44]. Moreover, the hypothalamic-pituitary-adrenal axis responded more strongly in the MOD condition with increased adrenocorticotropic hormone and cortisol concentrations, similarly to insulin-like growth factor 1 in NT, probably due to elevated heat production at a higher workload with increased stress. The hypothalamic-pituitary-thyroid axis demonstrated little to no change in its hormones from acute thermal or exercise stresses, and is consistent with previous findings [19]. Interestingly, free triiodothyronine demonstrated greater concentrations in SHIV compared to COLD. Although shivering and non-shivering thermogenesis are reversely correlated [45], our shivering protocol likely induced non-shivering thermogenesis as subjects, in the SHIV condition were cold-exposed prior to exercise for 40–120 min. Ribeiro et al. [46] suggested thyroid hormones-dependent pathways for heat production from uncoupling protein 1 activation in mitochondria found in brown adipose tissue. This would require a greater use in thyroid hormones. As this was observed in LOW only, the heat production from the MOD condition likely attenuated the need for non-shivering thermogenesis during the exercise portion of the trials.

### Leukocytes

The greater leukocytosis in NT observed in the present study, attributed mainly to increased lymphocytes, could be associated with greater catecholamine secretion, known to mobilize leukocytes out of the marginal pools in response to stress. β-adrenergic receptor expression on lymphocytes, particularly T and B cells and natural killer cells, are targeted by catecholamines and induce a cascade of events through the adenyl cyclase system for lymphocyte mobilization [47]. Although the degree by which the lymphocyte mobilization response may be determined by cell surface receptor density [25], the greater norepinephrine secretion in the SHIV condition compared to COLD did not seem to have the same effect on leukocyte response as it did in the NT condition. As increased testosterone levels, observed in NT, have been linked to T lymphocyte cells apoptosis [48], the difference in lymphocyte and total leukocytes in the NT and SHIV conditions remain unexplained and will require further investigation.

Cortisol and adrenocorticotropic hormone, part of the hypothalamic-pituitary-adrenal axis, generally respond by increasing due to an elevated inflammatory response and systematically act to suppress and control inflammation through changes in glucocorticoid receptor expression on lymphocyte cells. Fragala et al. [49] suggested a temporal role of the hypothalamic-pituitary-adrenal axis in modulating immune functions during one hour of exercise where despite increases in cortisol levels, glucocorticoid expression was lower in B-lymphocytes before increasing during recovery, indicating the possibility of greater suppression of an inflammatory response following exercise. Thereby, the increases in cortisol and adrenocorticotropic hormone concentrations in the NT condition may only have taken effect during later recovery, despite providing elements of an immuno-suppressive environment.

### Cytokines

Previous studies have examined a limited array of cytokines in response to exercise and thermal stress which consequently make it difficult to draw accurate interpretations of pro-inflammatory and anti-inflammatory immune responses [14], [37], [50]. We combined and indexed on a z score sixteen pro-inflammatory cytokines, three anti-inflammatory cytokines and four growth factors to examine immune responses during low and moderate-intensity exercise with environmental stress. Our approach indicated a clear shift of a pro-inflammatory response in NT and SHIV compared to COLD at moderate-intensity exercise. Interestingly, the anti-inflammatory response was not different between conditions, but followed a similar trend. IL-6 has been defined as both a pro- and anti-inflammatory myokine but was included in the pro-inflammatory group as its originally established and primary function. The increase in pro-inflammatory response in NT was mainly driven by changes in IL-1β, IL-7, IL-17, Rantes, IFN-γ, Eotaxin, IP-10, MIP-1β, and MCP-1 and in SHIV by IL-1β, IFN-γ, Rantes MIP-1β, and MCP-1. Shivering thermogenesis is described as involuntary asynchronized muscle contractions. From additional muscle work in the SHIV condition, we could have expected an increase in myokine secretion form the muscles influencing immune responses. However, no changes in Il-6, IL-8, IL-15 or IL-17 between SHIV and COLD were observed. Interestingly, the greater change in immune responses in SHIV occurred in the moderate exercise intensity but not in low intensity. This finding could be explained via the additional muscle damage and its associated inflammatory response. The mechanistic reasoning of exercise-induced inflammation is based on tissue damage from mechanical and chemical stimuli [51]. The pre-exercise cooling protocol induced deep tissue cooling, including skeletal muscle cooling, necessary to perform subsequent exercise [20]. A greater physical load at a fixed exercise intensity combined with cooled muscle can be indicative of greater muscle cell damage, increasing the inflammatory response in the MOD SHIV condition. The mechanisms explaining the inflammatory response to muscle damage is still poorly understood but recent evidence seem to indicate that neutrophils invade damaged sites and is responsible for the onset of inflammatory cytokines release [52], [53]. Although the granulocyte count (containing mainly neutrophils) in the MOD SHIV condition did not present any differences compared to other conditions, certain cytokines, associated with muscle cells damage [54] [55] [56], were elevated, including IL-1β and IFN-γ. This was also the case for a sub-cytokine group of chemokines, namely Rantes, MIP-1 β and MCP-1, also involved in tissue damage inflammatory response [57] and associated with increased sympathetic activity [58]. The greater concentration of norepinephrine in the SHIV *vs.* COLD conditions would explain this inflammatory shift. Granulocyte count in MOD was greater at the end of exercise in all conditions. The action of neutrophils of muscle cell lysis is well known but whether it relies more heavily on superoxide, hydrogen peroxide, myeloperoxide and/or other free radicals is unclear [59]. Since the z score indicated an increase in cytokine production in the MOD only in the SHIV condition, the implication of neutrophil-derived muscle damage and its associated production of cytokines cannot fully explain the present results.

Finally, associations between immuno-endocrine parameters (Tables S1, S2, S3) indicated a very limited amount of relationships in COLD as opposed to NT and SHIV. Cross et al. [60] observed a similar dissociation between variables when the rise in core temperature was abolished. The presence of other immune modulating mechanisms, with absence of thermal stress-related endocrine changes, would offset known associations.

### Methodological considerations

Although most studies examining immune changes from circulating venous concentrations of leukocytes and other markers, circulating concentrations of leukocytes represent a narrow 0.2–2% of total leukocyte mass [2], thus limiting the scope of interpretation in immune changes from various external stimuli. The examination of leukocytes and its sub-groups (lymphocytes, granulocytes and monocytes) did not offer specific modulations of lymphocytes subsets in T and B cells or in natural killer cells, the first line of defense of the immune system, which would have offered additional insights. The present study characterized the signaling of many hormones and immune measures during an acute exercise intervention in different environmental conditions. The novelty of this work includes the simultaneous examination of a large scale of responses from many hormonal axes, growth factors and cytokines modulated by cold exposure and shivering thermogenesis. Based on this work, future investigations need to tailor experimental protocols to individualize and target specific mechanisms of actions and functions of the present measures. Moreover, the possible long-term modulation of immune responses is needed to evaluate the influence of the cold on immune-related pathologies.

### Conclusion

In conclusion, our results demonstrated a reduced exercise-related immune response during exercise in the cold compared to a thermoneutral environment from a large array of endocrine and immune measures. However, we also observed an immuno-stimulatory effect from additional cooling, inducing shivering thermogenesis, compared to cold alone. Altogether, these results suggest a novel approach in partially inhibiting the acute systemic inflammatory response from exercise. Careful considerations need to be taken as additional cooling can reverse this response.

## Supporting Information

Table S1 **Pearson's correlation coefficients from exercising in NT condition.**
(DOCX)

Table S2 **Pearson's correlation coefficients from exercising in COLD condition.**
(DOCX)

Table S3 **Pearson's correlation coefficients from exercising in SHIV condition.**
(DOCX)
