# Supplementary material for: The Effects of Cold Exposure on Leukocytes, Hormones and Cytokines during Acute Exercise in Humans
Source: PLoS One. 2014 Oct 22;9(10):e110774. doi: 10.1371/journal.pone.0110774 (PMC4206434; doi:10.1371/journal.pone.0110774)
Supplement: Table S3 — Pearson's correlation coefficients from exercising in SHIV condition. (DOCX) [file pone.0110774.s003.docx]

S3. Pearson’s correlation coefficients from exercising in SHIV condition.

|  | *HR* | *T_core_* | *T_skin_* | *Epi* | *NE* | *TES_tot_* | *TES_bio_* | *SHBG* | *ACTH* | *COR* | *IGF-1* | *TSH* | *T_3_* | *T_3free_* | *T_4free_* |
| --- | --- | --- | --- | --- | --- | --- | --- | --- | --- | --- | --- | --- | --- | --- | --- |
| *Leukocytes* | NS | NS | NS | NS | NS | 0.747 | 0.696 | NS | NS | 0.550 | NS | NS | 0.566 | NS | NS |
| *Lymphocytes* | NS | NS | NS | NS | NS | NS | NS | NS | NS | NS | NS | NS | NS | NS | 0.574 |
| *Granulocytes* | NS | NS | NS | NS | NS | 0.761 | 0.708 | NS | NS | NS | 0.620 | NS | 0.557 | NS | NS |
| *Monocytes* | NS | NS | NS | NS | NS | NS | NS | NS | NS | NS | NS | NS | NS | NS | NS |
| *IL-1ra* | NS | 0.554 | NS | NS | NS | NS | NS | NS | NS | NS | NS | NS | NS | -0.676 | NS |
| *IL-1β* | NS | 0.538 | NS | NS | NS | NS | NS | NS | NS | NS | NS | NS | NS | -0.658 | NS |
| *IL-2* | NS | NS | NS | NS | NS | NS | NS | NS | NS | NS | NS | NS | NS | -0.785 | NS |
| *IL-4* | NS | NS | NS | NS | NS | 0.527 | NS | NS | NS | NS | NS | NS | NS | -0.673 | NS |
| *IL-5* | NS | NS | NS | NS | NS | NS | NS | NS | NS | NS | NS | NS | NS | -0.644 | NS |
| *IL-6* | NS | NS | NS | NS | NS | NS | NS | NS | NS | NS | NS | NS | NS | -0.830 | NS |
| *IL-7* | NS | NS | NS | NS | NS | NS | NS | NS | NS | NS | NS | NS | NS | -0.798 | NS |
| *IL-8* | NS | NS | NS | NS | NS | NS | NS | NS | NS | NS | 0.565 | NS | NS | -0.794 | NS |
| *IL-9* | NS | NS | NS | NS | NS | NS | NS | NS | NS | NS | 0.619 | NS | NS | -0.730 | NS |
| *IL-10* | 0.531 | NS | NS | NS | NS | NS | NS | NS | NS | NS | NS | NS | NS | -0.587 | NS |
| *IL-12* | NS | NS | NS | NS | NS | NS | NS | NS | NS | NS | NS | NS | NS | -0.690 | NS |
| *IL-15* | NS | NS | NS | NS | NS | NS | NS | NS | NS | NS | NS | NS | NS | -0.778 | NS |
| *IL-17* | NS | NS | NS | NS | NS | NS | NS | NS | NS | NS | 0.596 | NS | NS | -0.826 | NS |
| *Eotaxin* | 0.557 | NS | NS | NS | NS | NS | NS | NS | NS | NS | NS | NS | NS | NS | 0.539 |
| *FGF2* | NS | NS | NS | NS | NS | NS | NS | NS | NS | NS | NS | NS | NS | -0.852 | NS |
| *G-CFS* | NS | NS | NS | NS | NS | NS | NS | NS | NS | NS | NS | NS | NS | -0.548 | NS |
| *IFN-γ* | NS | 0.617 | NS | NS | NS | NS | NS | NS | NS | NS | NS | NS | NS | -0.589 | NS |
| *IP-10* | NS | NS | NS | NS | NS | NS | NS | NS | NS | -0.520 | NS | -0.533 | -0.521 | NS | NS |
| *PDGF* | NS | 0.648 | 0.649 | NS | NS | NS | NS | NS | NS | NS | NS | NS | NS | NS | NS |
| *MCP-1* | 0.573 | NS | NS | NS | NS | NS | NS | NS | NS | NS | NS | NS | NS | NS | NS |
| *MIP-1β* | 0.748 | NS | NS | NS | NS | NS | NS | NS | NS | NS | NS | NS | NS | -0.518 | NS |
| *Rantes* | NS | NS | NS | NS | NS | NS | NS | NS | NS | NS | NS | NS | NS | NS | NS |
| *VEGF* | NS | NS | NS | NS | NS | NS | NS | NS | NS | NS | 0.658 | NS | NS | -0.731 | NS |

HR, heart rate; t_core_, core temperature; t_skin_, skin temperature; Epi, epinephrine; NE, norepinephrine; TES_bio_, bioavailable testosterone, ACTH, adenocorticotropic hormone; IGF-1, insulin-like growth factor-1; TSH, thyroid-stimulating hormone; T_3_, triiodothyronine; T_3free_, free triiodothyronine; T_4free_, free thyroxine; IL, interleukin; FGF2, basic fibroblast growth factor; G-CSF; granulocyte colony-stimulating factor; IFN- γ; interferon gamma; IP-10, interferon gamma-induced protein 10; PDGF, platelet-derived growth factor; MCP-1, monocyte chemotactis protein-1; MIP-1β, macrophage inflammatory protein 1β; VEGF, vascular endothelial growth factor. NS, non-significant. Correlation coefficients are significant at *p* ˂ 0.05.
